# The Role of the Signaling Pathways Involved in the Protective Effect of Exogenous Hydrogen Sulfide on Myocardial Ischemia-Reperfusion Injury

**DOI:** 10.3389/fcell.2021.723569

**Published:** 2021-08-30

**Authors:** Shuangyu Lv, Xiaotian Li, Shizhen Zhao, Huiyang Liu, Honggang Wang

**Affiliations:** Henan International Joint Laboratory of Nuclear Protein Regulation, School of Basic Medical Sciences, Henan University, Kaifeng, China

**Keywords:** hydrogen sulfide, myocardial ischemia/reperfusion injury, apoptosis, signaling pathways, antioxidant

## Abstract

Ischemia/reperfusion (I/R) injury refers to the functional and structural changes in the process of blood flow recovery after ischemia. In addition to ischemia, the blood flow recovery can also lead to very harmful damage, such as the obvious cell swelling and the irreversible cell necrosis. I/R injury is related with many diseases, including myocardial I/R injury. Myocardial I/R injury refers to the aggravation of ischemic myocardial tissue injury due to sudden disorder of blood circulation. Although there are many studies on myocardial I/R injury, the exact mechanism is not fully understood. Hydrogen sulfide (H_2_S), like carbon monoxide and nitric oxide, is an important gas signal molecule. It plays an important role in many physiological and pathological processes. Recent studies indicate that H_2_S can improve myocardial I/R injury, however, its mechanism is not fully understood, especially the involved signal pathways. In this review, we summarize the related researches about the role of the signaling pathways involved in the protective effects of exogenous H_2_S on myocardial I/R injury, so as to provide theoretical reference for the future in-depth researches.

## Introduction

**FIGURE 1:**
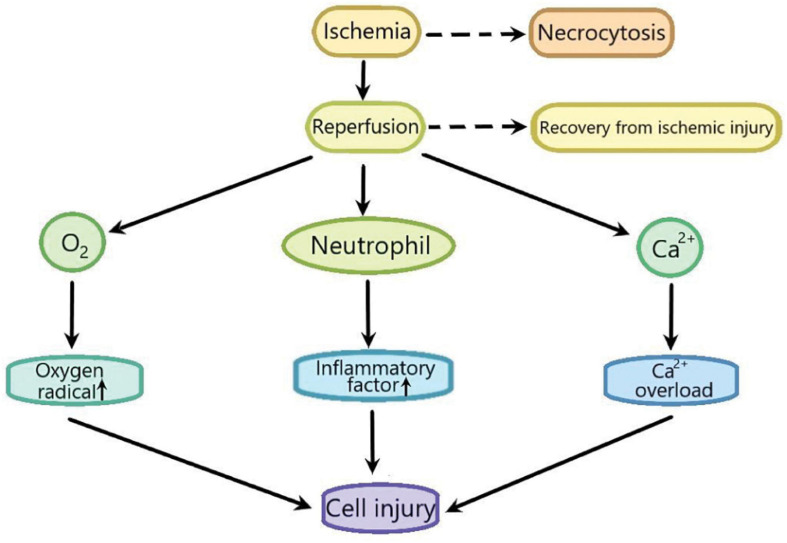
The sketch of the process of ischemia-reperfusion injury. Ischemia leads to cell necrocytosis. Reperfusion induces a large amount of Ca^2 +^ influx to lead Ca^2+^ overload which causes the cell injury. Reperfusion also induces the production of excessive oxygen free radicals, promotes the accumulation of pro-inflammatory factors such as neutrophils, and finally aggravates cell injury.

Ischemia/reperfusion (I/R) injury is used to describe the functional and structural damages which become apparent when the blood flow is restored after a period of ischemia. In addition to the ischemia, the recovery of blood flow can leads to potentially very harmful effects, such as the cell necrosis, the notable cell swelling, and the uneven blood flow of all parts of the recovered tissues (Oliveira et al., 2018; Soares et al., 2019). I/R injury is composed of two important events. Ischemia, the first important event, is the limitation of the blood supply to the organ, usually due to an embolus blocking the blood supply of an artery. The second important event is reperfusion, that is, the restoration of blood flow and reoxygenation in the affected ischemic area, which may further leads to the excessive tissue deterioration and trigger the destructive inflammatory response (Yan et al., 2020; Figure 1). I/R injury plays an important role in many diseases, such as heart diseases (Deguchi et al., 2020), brain diseases (Zhong et al., 2012), and liver diseases (Lin et al., 2020). Myocardial ischemia is a common phenomenon of coronary heart diseases. Myocardial I/R injury refers to the phenomenon that the injury of ischemic myocardial tissue becomes more serious due to the sudden disturbance of blood circulation (Lindsey et al., 2018). Although the researches on myocardial ischemia-reperfusion injury has been very extensive (Boag et al., 2017; Russo et al., 2017; Mokhtari-Zaer et al., 2018), its exact mechanism has not been fully understood.

**FIGURE 2:**
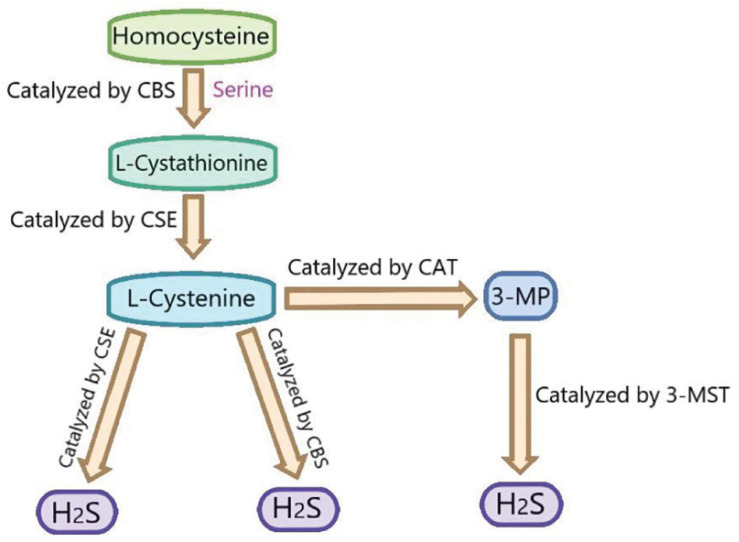
The summary of the production of endogenous H_2_S. CBS, cystathionine-beta-synthase; CSE, cystathionine-gamma-lyase; 3-MST, 3-mercaptopyruvate thiotransferase; 3-MP, 3-mercaptopyruvate; CAT, cysteine aminotransferase.

Hydrogen sulfide (H_2_S), which has the smell of rotten eggs, is a toxic, colorless and corrosive gas. Structurally, it is a sulfur analog of water molecules and can be oxidized to elemental sulfur, sulfate (SO_4_^2–^), sulfur dioxide (SO_2_), and thiosulfate (S_2_O^3–^) (Murphy et al., 2019). H_2_S was regarded as an environmental toxin until it was found to be endogenous (Paul and Snyder, 2018). In recent years, H_2_S, along with nitric oxide (NO) and carbon monoxide (CO), is the intracellular signal transduction molecules. It has been found that the low concentration of H_2_S plays an vital role in the physiological process (Olas, 2015). Three enzymes have been found to catalyze the production of endogenous H_2_S: cystathionine β-synthase (CBS), cystathionine γ-lyase (CSE), and 3-mercaptopyruvate sulfurtransferase (3MST) (Mustafa et al., 2009; Li et al., 2011; Wallace and Wang, 2015). CBS catalyzed the β-substitution of homocysteine with serine to produce L-cystathionine. CSE catalyzes the elimination of α, γ- cysteine of cystathionine to produce cystine. Under the catalysis of CBS and CSE, cysteine can produce H_2_S through the β eliminate reaction. Aminotransferase catalyzes cystine to transfer amine to α- Ketoglutarate to form 3-mercaptopyruvic acid (3-MP). The sulfur of 3-MP was catalyzes by 3-MST to convert into H_2_S (Wang et al., 2020; Figure 2). It has been reported that H_2_S plays an important biological role in many human systems, such as respiratory system, cardiovascular system, endocrine system, nervous system, immune system, and gastrointestinal system (Gotor et al., 2019). In recent years, there are many studies on the effects of H_2_S on myocardial I/R injury. However, its mechanism is not fully clear, especially the involved signal pathways. Therefore, we summarize the relevant researches about the above aspects to provide theoretical references for the future in-depth researches.

## The Role of JAK2/STAT3 Signaling Pathway Involved in the Protective Effect of Exogenous H_2_S on Myocardial I/R Injury

The Janus kinase/signal transducer and activator of transcription (JAK/STAT) signaling pathway is activated by a variety of interferons and cytokines, and widely involved in the tumor signal transduction. JAK is activated by the bingding of cytokines to its specific receptors of the cell membrane, which induces STAT phosphorylation. The phosphorylated STAT binds to the specific DNA elements and promotes the gene transcription (Murray, 2007). The JAK/STAT signaling pathway has been reported to be invoved in the innate and adaptive immunity, cells proliferation, tissues growth, angiogenesis and protease expression, and angiogenesis (Kiu and Nicholson, 2012; Yu et al., 2014). The JAK2/STAT3 signaling pathway has been demonstrated to play an important role in many kinds of heart diseases (Geng et al., 2019; Zhang J. et al., 2020), including myocardial I/R injury (Yin et al., 2020; Zhang Y. et al., 2020, however, the relevant mechanisms are not fully understood. Heng Fei Luan and colleagues found that NaHS (a donor of H_2_S) postconditioning attenuated the rat myocardial I/R injury by improving systemic hemodynamics, reducing myocardial infarct size and inhibiting cardiomyocyte apoptosis. While AG-490, a JAK2 inhibitor, abolished the cardioprotective effect of exogenous H_2_S. The in depth researches showed that NaHS postconditioning increased the expression of p-STAT3 and bcl-2, and decreased bax expression in the rat heart with I/R injury, while AG-490 counteracted these changes. Therefore, it can be deduced that H_2_S postconditioning protected the rat hearts against I/R injury through JAK2/STAT3 signaling pathway (Luan et al., 2012). Rapamycin, an autophagy activator, has been reported to attenuate myocardial I/R injury by opening mitochondrial K_*ATP*_ channel (Das et al., 2012), suggesting K_*ATP*_ channel is vital in myocardial I/R injury. Exogenous H_2_S can improve myocardial injury through opening K_*ATP*_ channel (Ji et al., 2008; Sun et al., 2015; Zhong et al., 2010), therefore, the relationship between JAK2/STAT3 signaling pathway and K_*ATP*_ channel in the protective effects of H_2_S on myocardial I/R injury is worthy of further study. Studies revealed that STAT3 played the cardioprotection role through scavenging oxidants (Lei et al., 2019). Therefore, whether exogenous H_2_S can alleviate myocardial I/R injury by eliminating oxidants through JAK2/STAT3 signaling pathway remains to be studied.

## The Role of Nrf2 Signaling Pathway Involved in the Protective Effect of Exogenous H_2_S on Myocardial I/R Injury

Nrf2 [nuclear factor (erythroid-derived 2)-like 2] is a transcription factor and is inhibited by interacting with the redox sensitive protein Kelch-like ECH-associated protein 1 (Keap1). Nrf2 is the main regulator of a group of antioxidant response elements containing cell protective genes induced in the stress response. It has been reported that H_2_S improves diabetes-accelerated atherosclerosis by suppressing oxidative stress *via* Keap1 sulfhydrylation at Cys151 to activate Nrf2 signaling (Xie et al., 2016). H_2_S also alleviates doxorubicin-induced myocardial fibrosis through inhibiting oxidative stress and apoptosis *via* Keap1-Nrf2 (Li Y. et al., 2021). Extracellular signal-regulated kinase (ERK), also known as MAPK, plays vital role in the proliferation, differentiation, and survival (Rai et al., 2019). It has been reported that H_2_S protects H9C2 cardiac cells against high glucose-induced injury *via* p38 MAPK and ERK1/2 pathways (Xu et al., 2013). So far, there are few reports on H_2_S improving myocardial I/R injury by activating Nrf2. The results of Peake et al. (2013) showed that the activities of three H_2_S producing enzymes (CBS, CSE, and 3-MST) in the heart of diabetic mice were reduced. The levels of free H_2_S and sulfane sulfur also were notably decreased in the heart and the blood of diabetic mice. Treatment with H_2_S in the form of sodium sulfide (Na_2_S) 24 h before myocardial ischemia (Na_2_S precondition) or 7 days before myocardial ischemia (Na_2_S 7d precondition) could notably reduce rat diabetic myocardial I/R injury by decreasing infarct size. Moreover, the myocardial protective effect of Na_2_S 7d precondition was better than that of Na_2_S PC. Pretreatment with Na_2_S decreased the oxidative stress and the apoptosis induced by myocardial I/R injury through reducing lipid peroxidation levels and cleaved caspase-3, respectively, suggesting that Na_2_S improve rat myocardial I/R injury by inhibiting oxidative stress and the apoptosis. The mechanism researches revealed that Na_2_S pretreatment activated Nrf2 signaling in rat diabetic myocardium with I/R injury, while diabetes impaired Nrf2 signaling. NQO1 and HO-1 are important antioxidants. Na_2_S 7d PC increased the expression of NQO1 and heme oxygenase-1 (HO-1) by promoting Nrf2 binding to the promoter of NQO1 and HO-1. Bach1, a known HO-1 transcription inhibitor, which impeded the Nrf2 binding to HO-1 promoter. The in depth researches showed that Na_2_S 7d PC could upregulate HO-1 expression by promoting the ERK1/2-dependent removal of Bach1 from the nucleus. Collectively, exogenous H_2_S improved diabetic myocardial I/R injury by upregulating the expression of NQO1 and HO-1 through activating Nrf2 signaling pathway in an ERK-dependent manner, which needs to be further confirmed with the inhibitor of Nrf2 signaling pathway (Peake et al., 2013). At present, there are few studies on the simultaneous activation of Nrf2 and ERK by H_2_S. Therefore, it is necessary to further study the mechanism of H_2_S activation of Nrf2/ERK. Increasing the level of H_2_S in cardiomyocytes is a potential strategy to reduce myocardial I/R injury in the setting of diabetes.

## The Role of SIRT1/PGC-1α Signaling Pathway Involved in the Protective Effect of Exogenous H_2_S on Myocardial I/R Injury

The silent information regulator of transcription 1 (SIRT1) is a highly conserved NAD^+^-dependent protein deacetylase, which deacetylates downstream peroxisome proliferator-activated receptor-γ co-activator-1α (PGC-1α) to promote its activity (Li et al., 2016; Wang et al., 2018). The SIRT1/PGC-1α signaling pathway has been demonstrated to participate in the regulation of many pathological processes related to cell survival, oxidative stress, intestinal homeostasis, and anti-aging (Hasegawa et al., 2008; Li et al., 2014; Wei et al., 2014). There are growing evidences that SIRT1/PGC-1α signaling pathway is involved in myocardial I/R injury (Tang et al., 2019; Tian et al., 2019; Wang et al., 2019), however, the related mechanism is not fully clear. The results of Ming Zhu Hu and colleagues showed that H_2_S postconditioning alleviated the rat hearts I/R injury by improving hemodynamic parameters, reducing the myocardial ischemia size, inhibiting the myocardial enzyme release, increasing ATP and superoxide dismutase (SOD) levels, and decreasing malondialdehyde (MDA) level. Exogenous H_2_S also upregulated the expression of SIRT1 and PGC-1α in the rat heart with I/R injury. EX-527, a selective SIRT1 inhibitor, reversed the above changes induced by H_2_S, suggesting that SIRT1/PGC-1α signaling pathway mediated the protective effect of exogenous H_2_S on myocardial I/R injury (Hu et al., 2016). The entry of SIRT1 into the nucleus is necessary for its cytoprotective effects against oxidative stress (Tanno et al., 2010), which may be invoved in the protective effect of H_2_S on myocardial I/R injury. The in depth research showed that in rat cardiomyocyte, I/R induced SIRT1 out of the nucleus and this was reversed by exogenous H_2_S, which was the mechanism of exogenous H_2_S activation of SIRT1/PGC-1α signaling pathway. Collectively, Exogenous H_2_S improves myocardial I/R injury in rats by activating SIRT1/PGC-1α signaling pathway (Hu et al., 2016). Studies have shown that high levels of NAD^+^, peroxisome proliferator activated receptor (PPAR), FOXO family transcription factors, and ubiquitination can regulate SIRT1 activity (Brunet et al., 2004; Kalliora et al., 2019; Chen et al., 2020; Yu et al., 2020). Therefore, whether exogenous H_2_S can regulate SIRT1/PGC-1α signaling pathway by the above substances needs further study.

## The Role of PI3K/SGK1/GSK3β Signaling Pathway Involved in the Protective Effect of Exogenous H_2_S on Myocardial I/R Injury

Phosphatidylinositol-3-kinase (PI3K) is a group of plasma membrane associated lipid kinases, which consists of three subunits (Donahue et al., 2012). It is involved in regulating proliferation, cell growth, and survival (Lee et al., 2011). The serum and glucocorticoid induced kinase-1 (SGK1) is a serine/threonine kinase widely expressed downstream of PI3K. SGK1 is ubiquitously expressed in various cell types (Lang et al., 2006, 2009). GSK3β is a vital downstream target of SGK1 and has been reported to alleviate myocardium I/R injury by regulating autophagy (Aoyama et al., 2005; Zhai et al., 2011). Jiang et al. (2016) found that the expression of t-PI3K, p-PI3K, t-SGK1, and p-SGK1 were decreased, the p-GSK3β expression was increased in cardiomyocyte exposed to hypoxia/reoxygenation (H/R), which were reversed by exogenous H_2_S, suggesting that exogenous H_2_S activated PI3K/SGK1/GSK3β signaling pathway in cardiomyocyte with H/R injury. The H/R treatment of rat cardiomyocytes reduced cell viability, and aggravated cell injury by increasing LDH releasing, which were reversed by exogenous H_2_S. Further researches showed that autophagy was notably increased in cardiomyocytes exposed to H/R, which was reversed by exogenous H_2_S. The inhibition of PI3K with LY294002 (a PI3K inhibitor) or knocking down SGK1 with SGK1 siRNA promoted autophagy and inhibited the anti-autophagy, and cardioprotective effects of exogenous H_2_S. While blocking GSK3β by tws119 (a GSK3β inhibitor) has the opposite effect. Collectively, it can be induced that exogenous H_2_S alleviated myocardial I/R injury by inhibiting autophagy through activating PI3K/SGK1/GSK3β signaling pathway (Jiang et al., 2016). The relationship between H_2_S and PI3K/SGK1/GSK3β signaling pathway, as well as the relationship between autophagy and PI3K/SGK1/GSK3β signaling pathway, has been rarely studied, which need further study.

## The Role of JNK Signaling Pathway Involved in the Protective Effect of Exogenous H_2_S on Myocardial I/R Injury

The c-Jun N-terminal kinases (JNKs) is a member of the mitogen activated protein kinase (MAPK) family and regulate the cell responses to a variety of exogenous and endogenous damages, including reactive oxygen species (ROS), radiation, DNA damage, bacterial antigens, heat, and inflammatory cytokines. In particular, the JNK signaling regulates many important physiological processes including metabolism and tissue homeostasis, cell damage repair and cell death/survival, and affects the life span of organisms (Tafesh-Edwards and Eleftherianos, 2020). The JNK signaling pathway is reported to be involved in myocardial I/R injury (Li A. et al., 2021; Yang et al., 2019). Li and Xiao (2020) found that the pretreatment with NaHS increased the left ventricular diastolic pressure (LVDP) and the maximum rate of pressure rise/fall, and decreased the left ventricular end-diastolic pressure (LVEDP) in rats with myocardial I/R injury. In the ischemia rats, the perinuclear space increased gradually, the arrangement of fibers was disordered, and the damage of the mitochondrial cristae and membrane was aggravated, which was reversed by exogenous H_2_S. The above suggested that exogenous H_2_S notably improved myocardial I/R injury. The in-depth studies showed that in the cardiomyocytes with I/R jury, exogenous H_2_S also increased the endogenous H_2_S level and induced the activity of CSE, SOD and GSH-Px, inhibited the activity of SOD, and reduced the level of phosphorylated JNK2. This indicated that exogenous H_2_S may alleviate myocardial I/R injury through antioxidant and JNK signaling pathway (Li and Xiao, 2020), which need to be furthely conformed with the inhibitor of JNK signaling pathway. In addition, JNK signaling pathway is closely related to the oxidative stress (Yang et al., 2017; Chen et al., 2018; Xu et al., 2020), so whether exogenous H_2_S can improve myocardial I/R injury by inhibiting oxidative stress through JNK signaling pathway is worthy of further study.

## Conclusion

In this review, we summerized the signaling pathways involved in the protective effect of exogenous H_2_S on myocardial I/R injury as follows: (1) exogenous H_2_S postconditioning improves the rat myocardial I/R injury through JAK2/STAT3 signaling pathway; (2) exogenous H_2_S protected diabetic mouse myocardial I/R injury through activating Nrf2 signaling pathway in an ERK-dependent manner; (3) exogenous H_2_S alleviates myocardial I/R injury in rats by activating SIRT1/PGC-1α signaling pathway; (4) exogenous H_2_S alleviated myocardial I/R injury by inhibiting autophagy through activating PI3K/SGK1/GSK3β signaling pathway; and (5) exogenous H_2_S alleviates myocardial I/R injury through inhibiting JNK signaling pathway (Table 1).

**TABLE 1:** The summary of exogenous hydrogen sulfide improvements of myocardial I/R injury.

The protective mode of myocardial ischemia-reperfusion (I/R) injury	The mechanism of H_2_S protection against myocardial I/R injury	Experimental model	The mode of H_2_S action on myocardium
Exogenous H_2_S improves myocardial I/R injury through JAK2/STAT3 signaling pathway	Activating the JAK2/STAT3 signaling pathway	Rat myocardial I/R injury model	Postconditioning
Exogenous H_2_S protectes diabetic mouse myocardial I/R injury through activating Nrf2 signaling pathway in an ERK-dependent manner	Activating the Nrf2/ERK signaling pathway	Rat myocardial I/R injury model	Preconditioning
Exogenous H_2_S alleviates myocardial I/R injury in rats by activating SIRT1/PGC-1α signaling pathway	Activating the SIRT1/PGC-1α signaling pathway	Rat myocardial I/R injury model	Postconditioning
Exogenous H_2_S alleviated myocardial I/R injury by inhibiting autophagy through activating PI3K/SGK1/GSK3β signaling pathway	Inhibiting autophagy through activating PI3K/SGK1/GSK3β signaling pathway	The model of the neonatal rat cardiomyocytes exposed to hypoxia/reoxygenation	Preconditioning
Exogenous H_2_S alleviates myocardial I/R injury through inhibiting JNK signaling pathway	Inhibiting JNK signaling pathway	Rat myocardial I/R injury model	Postconditioning

Hydrogen sulfide is now regarded as the third kind of signal gas transmitter after NO and CO. It has a wide range of physiological and pathophysiological functions, including vasodilation, induction of angiogenesis, regulation of inflammatory response, regulation of glucose homeostasis, and regulation of neuronal activity. However, its role has not been fully studied. The mechanism of H_2_S in the process of myocardial I/R injury remains to be further elucidated. For example, in addition to the signal pathways mentioned in this manuscript, is there any other signal pathway involved in the above effects? Can high concentration of H_2_S aggravate myocardial I/R injury through the specific signaling pathways? In addition, the studies indicate that the cardioprotective effect of H_2_S is related to gender. Estrogen can regulates the production and release of H_2_S in cardiovascular cells to increase cell proliferation, cell migration, and vasodilation, which exert its cardiovascular protective effects (Teoh et al., 2020). The above related signal pathway mechanism needs to be clarified. H_2_S can improve myocardial inflammation in diabetic mice by inhibiting NLRP3 inflammasome (Jia et al., 2020), I/R can cause inflammatory injury of tissue (Zhou et al., 2014), so whether H_2_S can improve myocardial I/R injury by inhibiting NLRP3 inflammasome and the related signaling pathways deserve further study.

With the in-depth study of the signal pathway of H_2_S in the process of myocardial I/R injury, the use of H_2_S donor in the treatment of myocardial ischemia-reperfusion injury will become a very promising therapeutic strategy.

## Author Contributions

HW devised, wrote, and funded with the manuscript. SL wrote and funded with the manuscript. XL drew the figures. SZ and HL wrote the manuscript. All authors contributed to the article and approved the submitted version.

## Conflict of Interest

The authors declare that the research was conducted in the absence of any commercial or financial relationships that could be construed as a potential conflict of interest.

## Publisher’s Note

All claims expressed in this article are solely those of the authors and do not necessarily represent those of their affiliated organizations, or those of the publisher, the editors and the reviewers. Any product that may be evaluated in this article, or claim that may be made by its manufacturer, is not guaranteed or endorsed by the publisher.
